# Botulinum Toxin A for Oral Cavity Cancer Patients: In Microsurgical Patients BTX Injections in Major Salivary Glands Temporarily Reduce Salivary Production and the Risk of Local Complications Related to Saliva Stagnation

**DOI:** 10.3390/toxins4110956

**Published:** 2012-10-24

**Authors:** Corradino Bartolo, Di Lorenzo Sara, Moschella Francesco

**Affiliations:** Department of Surgical Oncology, Plastic Surgery Unit, University of Palermo, Italy; Email: bartolo.corradino@unipa.it (C.B.); dilsister@libero.it (M.F.)

**Keywords:** Botulinum toxin, salivary production, oral cancer, free flap complications, saliva-related complications, forearm free flap, oro-cutaneous fistula, salivary major glands

## Abstract

In patients suffering from oral cavity cancer surgical treatment is complex because it is necessary to remove carcinoma and lymph node metastasis (through a radical unilateral or bilateral neck dissection) and to reconstruct the affected area by means of free flaps. The saliva stagnation in the post-operative period is a risk factor with regard to local complications. Minor complications related to saliva stagnation (such as tissue maceration and wound dehiscence) could become major complications compromising the surgery or the reconstructive outcome. In fact the formation of oro-cutaneous fistula may cause infection, failure of the free flap, or the patient’s death with carotid blow-out syndrome. Botulinum injections in the major salivary glands, four days before surgery, temporarily reduces salivation during the healing stage and thus could reduce the incidence of saliva-related complications. Forty three patients with oral cancer were treated with botulinum toxin A. The saliva quantitative measurement and the sialoscintigraphy were performed before and after infiltrations of botulinum toxin in the major salivary glands. In all cases there was a considerable, but temporary, reduction of salivary secretion. A lower rate of local complications was observed in the post-operative period. The salivary production returned to normal within two months, with minimal side effects and discomfort for the patients. The temporary inhibition of salivary secretion in the post-operative period could enable a reduction in saliva-related local complications, in the incidence of oro-cutaneous fistulas, and improve the outcome of the surgery as well as the quality of residual life in these patients.

## 1. Introduction

In the majority of patients with oral cancer. Tumor excision and neck dissection must be followed by microsurgical reconstruction of the affected area. This radical surgical treatment associated with chemotherapy and radiotherapy improves the prognosis and the life expectancy of these often very young patients. For this reason the microsurgical reconstruction is really important and has to be safe and effective, in order to improve the quality of life of these unfortunate patients. Despite careful postoperative monitoring, systemic antibiotic therapy and careful nutrition, the incidence of local complications after these operations, is high. The percentage of wound dehiscence, infection and fistula formation in some reports is very high (up to 40%) [1,2,3].

In a small percentage of cases, complications can appear due to technical errors and incorrect surgical planning (for example an inappropriate choice of flap), but in most cases the main reason for local complications is saliva stagnation, caused by reduced clearance ability of the mouth, reduced capacity to swallow, and increased salivary secretion. In fact the saliva causes tissue maceration, wound dehiscence and bacterial contamination, which may often cause, on day five or six after the surgery, the formation of a dangerous oro-cutaneous fistula, that compromise the reconstruction and, sometimes, the life of the patient (carotid artery blowout syndrome). 

Infiltration of botulinum toxin in the major salivary glands was used for the first time (Bushara) in patients suffering from amyotrophic lateral sclerosis and other neurological disorders caused by muscarinic type cholinergic hyperactivity [4,5,6,7,8,9,10].

The authors, who had experience with botulinum toxin A (BTXA) for the aesthetic treatment of wrinkles, thought that infiltration of botulinum toxin A in the major salivary glands, in patients who require reconstructive microsurgery of the oral cavity by means of free flaps, could temporarily decrease salivary production up to 70% and then reduce of the incidence of local complications caused by the stagnation of saliva [11].

## 2. Results and Discussion

From January 2004 to June 2012, 43 patients suffering from oral cavity carcinoma underwent surgery; there are three phases of surgery: tumor excision, neck dissection (unilateral or bilateral) and microsurgical reconstruction with free flaps. 

Carcinoma was located in the tongue in 18 cases and in the floor of the mouth in 13 cases; it was disseminated widely in both locations in 12 cases. After wide radical surgery (excision of the area affected by the cancer and neck dissection) microsurgical reconstruction always followed. 

Different kinds of flaps were prepared: 14 ALT flaps, 16 forearm flaps, nine chimeric ALT-vastus lateralis, a double microsurgical free flap of rectus abdominis and a fibula flap in three cases.

**Figure 1:**
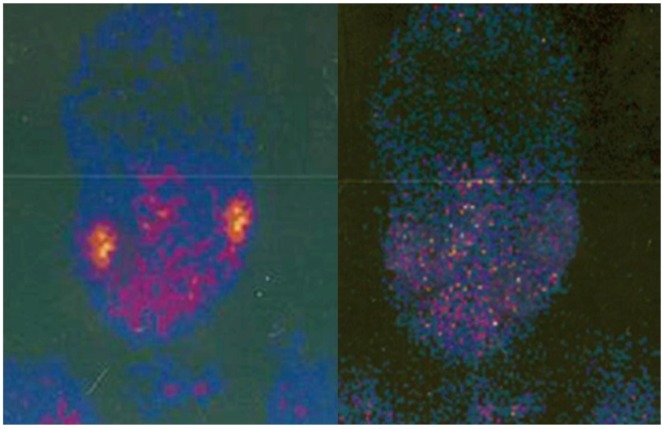
Left, pre-operative sialoscintigraphy; Right, sialography 15 days after btx injections.

All the patients, underwent sialoscintigraphy and a quantitative measurement of salivary secretion at the pre-operative period (Figure 1 left). 

The measurement of the salivary secretion was performed by setting four rollers of gauze (previously weighed using precision scales) for 2 min in the patient’s gingival fornix. After 2 min, every roller was weighed and the salivary secretion was quantified. 

Sialography before the surgery was normal in almost all patients and saliva production was higher than normal in just a few cases. 

**Figure 2:**
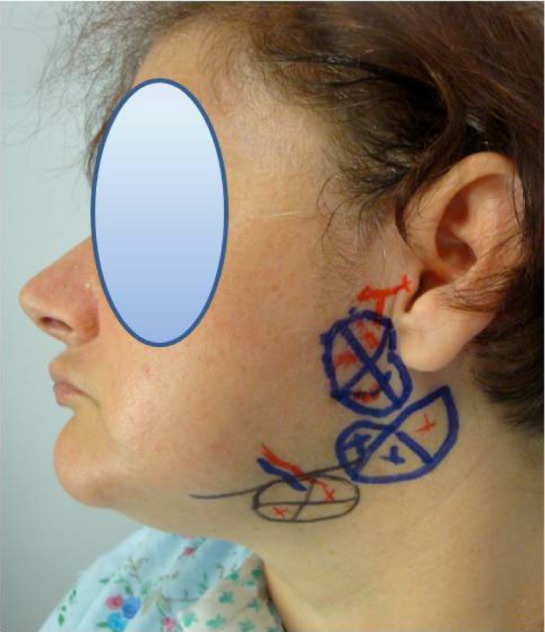
Sites of botox injections.

The infiltration technique is easy to perform. Four days before the surgery the authors injected the salivary glands with BTX (Figure 2).

First 100 U of BTX A (Botox, Allergan) was diluted in 1 cc of NaCl (0.9%). 

Then a 1 mL syringe with a 29 gauge needle was used. The major salivary glands were marked for the injection. All patients gave informed consent for this procedure. 

The superficial and deep lobes of the parotid gland were marked and injected; 3–4 injections per lobe. 

The injection points for the deep lobe are in a 3 cm-diameter-area located between the mastoid process and the mandibular ascending ramus (total of 15–20 U of toxin); three injections for the superficial lobe in a 3 cm-diameter-area located 1 cm under and in front of the tragus and on the back edge of the masseter muscle (15–20 U). Ten units of toxin were infiltrated for the submandibular glands (Figure 2). Of course, infiltration of the gland ipsilateral to the dissection of the neck is not necessary, since the gland is to be removed during surgery

The injections of the submandibular glands are not really important because in the majority of cases these glands are to be removed during the neck dissection. The total quantity of the infiltrated toxin was 80–100 U for each patient [8].

The greatest effect of the toxin was noticed between the fifth and eighth day after the injections (between the first and fourth days after surgery). 

The quantitative measurement of saliva was performed three and eight days after the infiltration using the method with gauze rollers in the gingival fornix. This method showed a 50% reduction of secretion on the third day after the injection and a 70% reduction on the eighth day (four days after surgery).

In 2010 the authors published the results of this evaluation on a smaller sample of patients (20 patients), and showed that while the mean salivation before BTX infiltrations was 0.24 gr/2 min, the mean salivation eight days after BTX injections was 0.08 gr/2 min. 

The data are statistically significant and prove that BTX injection temporarily reduces the saliva production (student’s *t* test 4; *p* > 3,883; α < 0,001; gdl 19) [11].

Sialoscintigraphy with Tc 99, repeated 15 days after the toxin infiltration (Figure 1, right) showed a 90% reduction of the function of the parotid and submandibular glands (clearly in patients in which the submandibular glands were not excised during the surgical operation), with a total of 80% reduction of salivary secretion. A basal secretion of saliva remains in all cases because of the presence of the minor salivary glands and due to the inefficacy of the toxin near the portion of the salivary glands with adrenergic innervations. 

This basal secretion was shown to be important because it protected the patients against oral cavity infections without causing or aggravating xerostomia or dry mouth caused by radiotherapy.

The most important side effect related to the botulinum toxin injections is symptomatic xerostomia. This side effect must be indicated in the specific patient’s consent, because after radical surgery almost all patients are submitted to radiotherapy. On the 45th day after the toxin infiltration, all the patients again underwent a quantitative measurement of salivary secretion which returned to a normal level in 36 of the patients. No complications due to toxin infiltration were noticed. During the first week after surgery five patients had local complications, due to fungus infection, showing dehiscence of sutures (three patients) and oro-cervical fistula formation (two patients, 4.6%). 

## 3. Conclusions

In this series the reduction of saliva secretion after botulinum toxin injections caused a reduction in the incidence of local saliva-related complications, improving wound healing, producing faster recovery, faster return to natural oral feeding and a shorter post-operative period. The effect of the drug is temporary and the risk of side effects is low. The technique of injections is easy and well tolerated by patients. 

In patients included in this study the percentage of complications related to salivary stagnation appears to be considerably reduced compared to other data in the literature. A precise valuation cannot be done because it is difficult to determine which complication can be attributed exclusively to the salivary stagnation and which not. Even though the study did not consider a control group, because of the small number of patients each year, it was possible to estimate and compare the post-operative complications in patients who had had a surgery in our department between the years 2000 and 2004. In fact during this period the botulinum toxin injections in major salivary glands were not carried out. There were only 12 patients who were operated on between 2000 and 2004 for oral cavity carcinoma with microsurgical reconstruction and presented similar characteristics to the group of 43 patients operated on between the years 2004 and 2012. In nine of twelve cases the flap used for reconstruction was the antebrachial free flap, in two cases ALT and only one case of fibular flap. There were six cases with local complications. In three cases a partial dehiscence of oral surgical suture appeared in the correspondence of the passage point of the vascular pedicle through the oral cavity floor, with formation of oro-cutaneous fistula which healed spontaneously. In another two cases the surgical suture dehiscence caused total detachment of the flap and oro-cervical fistula formation. In both cases another two minor operations were needed for revision of the sutures, debridement and closure of the fistula 15 and 30 days after surgery. In one case an infection in correspondence of the oral cavity floor under the chinese flap caused a thrombosis of the vascular pedicle with necrosis of the microsurgical flap frankly evident seven days after surgery. The removal of the flap was necessary and the reconstruction with pectoralis a major myocutaneopus flap was carried out. Reconstruction with a pectoralis major flap used to be more frequent previously, while these days it represents a rescue procedure, especially when another microsurgical intervention is controindicated. Even the pectoralis flap reconstruction is not free of complications related to salivary stagnation. 

This experience demonstrates that the toxin enables a reduction in local saliva-related complications, but it is difficult to determine the real rates of reduction of local complications in this series because of their multifactorial etiology. Statistical analysis of these data suggests that botulinum toxin in salivary glands is highly effective in reducing salivation and probably the related complications in the oral cavity (*p* < 0.001). The temporary inhibition of salivary secretion at the post-operative stage could enable a reduction in both saliva-related local complications and the incidence of oro-cutaneous fistulas. It could improve wound healing producing a faster recovery, a faster return to natural oral feeding and a shorter postoperative period, thus improving the outcome of the surgery and ultimately the quality of residual life of these patients.
